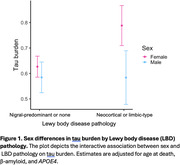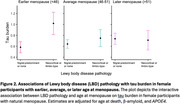# Synergism between Lewy body and Alzheimer’s disease pathology differs by sex

**DOI:** 10.1002/alz.093345

**Published:** 2025-01-03

**Authors:** Madeline Wood Alexander, Lisa L. Barnes, Julie A. Schneider, Kaitlin B. Casaletto, Jennifer S Rabin

**Affiliations:** ^1^ University of Toronto, Toronto, ON Canada; ^2^ Sunnybrook Research Institute, Toronto, ON Canada; ^3^ Rush Alzheimer’s Disease Center, Rush University Medical Center, Chicago, IL USA; ^4^ Rush Alzheimer's Disease Center, Rush University Medical Center, Chicago, IL USA; ^5^ Memory and Aging Center, UCSF Weill Institute for Neurosciences, University of California, San Francisco, San Francisco, CA USA; ^6^ Harquail Centre for Neuromodulation, Sunnybrook Research Institute, Toronto, ON Canada

## Abstract

**Background:**

Parkinson’s disease and dementia with Lewy bodies are more common in men, while Alzheimer’s disease (AD) is more common in women. Lewy body disease (LBD) and AD pathologies are highly comorbid and may be mechanistically linked. It remains unclear whether LBD and AD co‐pathology manifest differently across sexes. Here, we investigated sex differences in associations of LBD with β‐amyloid and tau. We also tested whether age at menopause modifies the relationship between LBD and AD pathology in women.

**Method:**

We used data from autopsied participants in the Religious Orders Study, Memory and Aging Project, and Minority Aging Research Study cohorts. Immunohistochemistry was used to quantify LBD stage, β‐amyloid, and tau. To capture cortical LBD pathology, LBD stage was dichotomized as neocortical‐type/limbic‐type vs. nigral‐predominant/none. Linear regressions tested interactive effects of 1) sex and LBD on AD pathology in the entire sample and 2) age at menopause and LBD on AD pathology among *female participants who reported natural menopause, adjusting* for age at death and *APOE4*.

**Results:**

We included *N* = 1,798 participants (mean (*SD*) age at death = 89.5 (6.74) years, *N* (%) female = 1,225 (68.1)). Between sexes, men exhibited more LBD pathology after adjusting for β‐amyloid and tau (β = 0.31, *p* = .02), while women showed a greater burden of both β‐amyloid (β = ‐0.10, *p* = .002) and tau (β = ‐0.08, *p* = .01) after adjusting for LBD. There was an interaction between sex and LBD on tau but not β‐amyloid, such that women with LBD had more tau than men with LBD after adjusting for β‐amyloid (male sex*β‐amyloid: β = 0.03, *p* = .7; male sex*tau: β = ‐0.16, *p* = .03; Figure 1). In women with natural menopause, age at menopause modified the association of LBD with tau, such that women with earlier menopause had greater tau burden in the presence of LBD (β = ‐0.03, *p* = .003; Figure 2). Post hoc analyses showed that the interactions between 1) sex and LBD on tau and 2) age at menopause and LBD on tau were driven mainly by neocortical‐type LBD and not limbic‐type LBD.

**Conclusion:**

LBD pathology might have a more pronounced impact on tau vulnerability in women than men. Female hormonal processes may further modify the relationship between LBD pathology and tau.